# Reducing Needle Stick Injuries in Healthcare Occupations: An Integrative Review of the Literature

**DOI:** 10.5402/2011/315432

**Published:** 2011-03-31

**Authors:** Lin Yang, Barbara Mullan

**Affiliations:** School of Psychology, University of Sydney, Brennan McCallum Building A18, NSW 2006, Australia

## Abstract

Needlestick injuries frequently occur among healthcare workers, introducing high risk of bloodborne pathogen infection for surgeons, assistants, and nurses. This systematic review aims to explore the impact of both educational training and safeguard interventions to reduce needlestick injuries. Several databases were searched including MEDLINE, PsycINFO, SCOPUS, CINAHL and Sciencedirect. Studies were selected if the intervention contained a study group and a control group and were published between 2000 and 2010. Of the fourteen studies reviewed, nine evaluated a double-gloving method, one evaluated the effectiveness of blunt needle, and one evaluated a bloodborne pathogen educational training program. Ten studies reported an overall reduction in glove perforations for the intervention group. In conclusion, this review suggests that both safeguard interventions and educational training programs are effective in reducing the risk of having needlestick injuries. However, more studies using a combination of both safeguards and educational interventions in surgical and nonsurgical settings are needed.

## 1. Introduction

Needle-stick injuries are an important and common occupational injury among healthcare workers. In a UK report, 37% of nurses reported that they have sustained a needle-stick injury at some stage during their career [1]. In Australia, the rate of reported needle-stick injuries is 1 in 5 occupied beds per year which equates to an annual sharps-related injuries incidence of 47,000 [2]. 

According to the policy of the NHS in the UK, it is compulsory when staff sustain a needle-stick injury to report the incident [3]. However, evidence from the US suggests that more than half of all sharps-related injuries are not reported [1]. Poor reporting of sharps-related injuries reveals a failure to appreciate the potential consequences of such injuries [4]. Rates of detection are also low, for example, only 11% of glove perforations were detected by the physician in a study investigating the use of blunt needles during obstetrical laceration repair surgeries [5]. 

Needle-stick injuries have been widely recognised as a source of exposure to bloodborne pathogens for workers in healthcare occupations [6]. There are more than 20 bloodborne pathogens that can be transmitted from contaminated needles or sharps, including hepatitis B (HBV), hepatitis C (HCV), and human immunodeficiency virus (HIV; NHS Employees, 2005). The risk of transmission of HIV following a hollow needle injury is approximately 0.3%, compared with 3% for HCV and 30% for HBV [7]. Worldwide, more than 100 healthcare workers have contracted HIV from work-related needle-stick injuries and many thousands have contracted HBV or HCV [2]. 

Due to the recognised risk of needle-stick injuries, safeguards have been put in place to attempt to lessen the risk of injury. These include the policy of universal precautions and needleless systems to connect with intravenous tubing [5]. Several strategies have been adopted for use in the healthcare setting, including double gloving, having a neutral zone in which to pass sharps, and the use of blunt tip needles [8]. Wearing two pairs of gloves is a practice which protects healthcare workers from patients' blood and body fluids. A recent study found that in 82% of cases when the outer glove was perforated, the inner gloves had been found to protect the wearer's hand from contamination [9]. However, discomfort, restriction of dexterity and impaired sensation of touch were reported to outweigh the benefits afforded by double gloving. Blunt needles require much greater force to induce glove perforation than sharp needles which can protect surgeons from needle-stick injuries [4]. Recently introduced guidelines for the prevention of sharps injuries in healthcare in Australia include, but do not mandate, access to and the use of safe engineered devices [10]. In the USA, the centres for disease control and prevention recommend the use of universal precautions to minimise exposure to bloodborne pathogens [11]. Knowledge and adoption of universal precautions were also associated with a significantly reduced risk of occupational exposure in a cohort of Australian nurses [12]. All of the practices aim to avoid direct contact of healthcare personnel with organic material of the patients.

Nevertheless, the health technology supply industry has not successfully developed cost-effective devices by which to protect the healthcare worker and so are not routinely used in the developing countries such as China despite the higher prevalence of bloodborne pathogens [13]. Using needle protective devices or double gloving may be cost effective in the long term [14], with a additional 5% cost and 6% cost saving having been reported [15]. However, there is a need for undertaking more cost-effective analysis to determine further validation. 

Generally, interventions around healthcare occupational injuries fall into two major categories, those using double-gloving or blunt needles and those providing educational messages. Interventions using double-gloving or blunt needles are often implemented in different hospital departments or in different operations. Evaluations are generally designed as randomised control trials, and target surgical departments or emergency medical departments. In contrast, educational interventions provide information about the negative consequences of healthcare occupational injuries or provide strategies to change attitudes to the occupational injuries. These programs are generally targeted at students and evaluated in before-and-after designs. 

Both types of intervention have costs associated with them, particularly in the interventions using new devices such as double gloving and blunt needles that need more funding [16]. Along with the flow of evidence-based interventions and the cost-effective principle, it is important that interventions are rigorously evaluated. Although a number of studies into needle-stick injuries interventions have been conducted in recent years, few have been systematically analysed [6]. 

The purpose of this paper is to conduct a rigorous and integrative review of interventions designed to decrease healthcare occupational injuries. To our knowledge, this is the first integrative review of the effectiveness of interventions on healthcare occupational injuries during the past 10 years. The previous review found a decrease in glove perforations when double gloves or combinations of gloves were used [6]. The importance of the study is that not only does it recognise the effective strategies on decreasing healthcare occupational injuries, but also recognises the shortage of evaluated studies. 

## 2. Methods

### 2.1. Design and Search Methods

**Figure 1:**
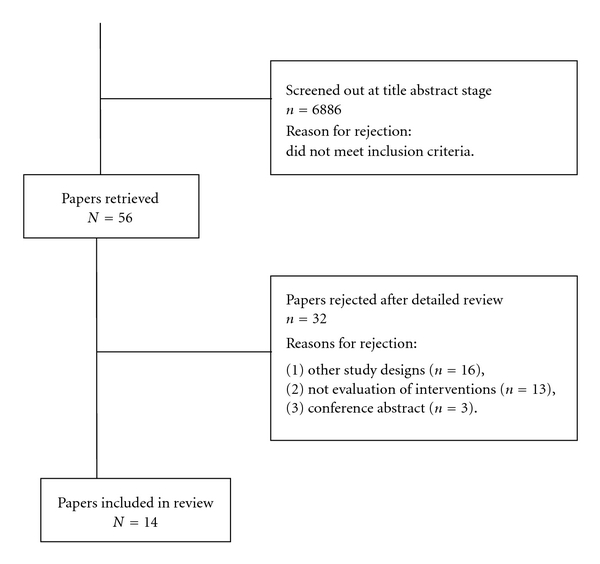
Selection of papers for the review.

Using an integrative approach to literature searching, searches were conducted in five different databases, that is, MEDLINE, PsycINFO, SCOPUS, CINAHL, and Sciencedirect. The terms “glove perforation,” “Needle-stick injuries,” “reducing percutaneous injuries,” “gloving tears,” “reducing sharp injuries,” and “occupational exposure in healthcare” were used in the initial search in all databases. All of the six terms were valid in MEDLINE, the first four terms were valid in Sciencedirect, and glove perforation produced different results in SCOPUS in addition to the results obtained from the other databases. “Needle-stick injuries,” “percutaneous injuries,” and “occupational exposure” were valid in PsycINFO, and “glove perforation” and “needle-stick injuries” in CINAHL. Six thousand nine hundred and fifty-six articles were screened for inclusion (see Figure 1 for details). PRISMA guidelines on undertaking reviews were followed, with reference to the evaluative checklist for reviewing outcome measures [17].

### 2.2. Search Outcome and Quality Appraisal

Only studies evaluating a needle-stick injury intervention, and deliberately designed to contain both a study and a control group within the past 10 years were included. Both studies using new safeguards and providing educational training were reviewed. There were no exclusion criteria regarding the types of participants, duration of intervention, or method of outcome measurement. 

Studies were rejected if they were not published in a peer-reviewed journal or if the text was not in English. A study was rejected if it did not meet the inclusion criteria determined from the title and abstract during screening. The full text of the study was reviewed for further evaluation if the title or abstract met the inclusion criteria or could not be rejected with certainty. Tables 1 and 2 were developed to specify the key information about each reviewed study.

## 3. Results

### 3.1. Study Selection

The search yielded 6942 bibliographic records and fourteen studies met the inclusion criteria [8, 9, 18, 19, 22–24, 26–28] (see Figure 1 for details). No studies were reported in multiple papers.

### 3.2. Study Characteristics

#### 3.2.1. Study Design

Of the fourteen studies, 10 were randomised control trials of interventions [5, 18–24, 26, 27]. Two studies used a cohort design with no randomisation of participants into conditions [8, 28]. Participants in these cohort studies choose the gloving types at their own discretion. One study was designed in a prospective randomised manner [9]. In this study, the surgeons were randomised to either the study group or the control group by sealed envelopes, and, then, the first assistant was automatically allocated into the opposite condition. The remaining study was a quasiexperimental design using randomisation procedure for groups rather than for each participant [25]. This study compared outcomes preintervention and postintervention between the intervention and the control group.

#### 3.2.2. Types of Intervention

Of the fourteen studies, ten studies evaluated the use of double gloves in preventing needle-stick injuries [8, 9, 18, 19, 22–24, 26–28]. This included eight studies evaluating the use of double gloves [8, 9, 18, 19, 22, 23, 26, 28], and two studies evaluating variations of double gloving [23, 24, 27]. Three studies evaluated the use of blunt needles in preventing needle-stick injuries [5, 20, 21]. The remaining study compared a bloodborne pathogen educational training with a standard education [25]. 

Thirteen out of the fourteen studies were conducted in departments of surgery or of emergency medicine and focused on different operations [5, 8, 9, 18–24, 26–28]. Nine of these studies focused on specific surgical procedures, including laparotomy surgery, nonemergent Caesarean delivery, obstetric laceration, elective gastrointestinal procedures, gynaecologic surgery, visceral surgical procedure, arch bar placement for intermaxillary fixation, episiotomy repair after vaginal delivery, orthopaedic and trauma surgery [5, 8, 18–23, 27]. Four studies included general surgeries, which included surgical procedures lasting more than one hour [9, 24, 26, 28]. The remaining study was conducted in a department of nursing [25].

### 3.3. Study Populations

Thirteen studies recruited participants from hospitals [5, 8, 9, 18–24, 26, 28]. Of these, five recruited surgeons and assistants [5, 9, 21, 24, 28], six only recruited surgeons [8, 19, 20, 22, 23, 27] and two recruited the whole operation team including scrub nurses [18, 26]. The final study recruited students in a department of nursing [25]. The subjects in this study had completed 3 years of academic work and 3 months of clinical practice. 

The fourteen studies used different methods to determine sample size. Six studies used individual participants as a unit of measurement, four of these counted “patients treated” as the sample, the sample size varied from 42 to 438 [5, 20, 21, 27]. One study used the number of surgeons who participated in the study as the sample size with 170 surgeons included [19], and the remaining study used the 106 students who participated in the study as the sample size [25]. Four studies used the number of procedures observed as the sample size which varied from 66 to 885 [9, 22–24]. Four studies used the number of pairs of gloves used in the study as the sample size which varied from 300 to 1000 pairs [8, 18, 26, 28]. The follow-up period varied from 2 months to 21 months with five studies not reporting the study duration. Only one study reported the mean age (19 years old) [25]. No study reported the gender proportion of participants.

### 3.4. Outcome Measures

In terms of outcome measures, the rate of glove perforation was assessed in all studies [5, 8, 9, 18–28]. Other outcome measures included detection rate of glove perforation (5 studies), evaluation of the devices used (4 studies), the relationship between glove perforation and the job level of surgeons (3 studies), the relationship between glove perforation and duration of the operation (8 studies), the relationship between glove perforation and surgical types (3 studies), frequency of glove perforations by position on surgical team (3 studies), changes in knowledge and self-reported universal precautions behaviour, observed adherence to universal precautions, and self-reported needle-stick/sharp injuries (1 study). For the purpose of this review, rate of glove perforation, detection rate of glove perforation, evaluation of devices used, changes in knowledge self-reported universal precautions behaviour, and observed adherence to universal precautions were analysed.

### 3.5. Study Quality

All fourteen studies contained both an experimental group and a control group. Of the fourteen trials, ten studies reported an adequate randomisation method, all using a cluster randomisation procedure [5, 18–24, 26, 27]. Two cohort studies did not use a randomisation procedure to allocate participants into conditions [8, 28]. Participants in these studies choose the gloving types at their own discretion. In one study, only surgeons were randomised to conditions whereas assistants were automatically allocated into the opposite condition [9]. The remaining study used randomisation procedure for groups rather than for each participant [25]. 

Outcomes in thirteen of the studies were measured by a water-leak test method, in one study gloves were filled with air then put in water [21]; another used both air and water-leak techniques [5]. Five studies tested unused gloves as controls to test for preexisting minor perforations [5, 9, 18, 19, 21]. One study did not test the glove perforation rate but used questionnaires and direct observation to collect data [25]. Direct observation was used in only three studies excluding the one which did not aim to measure the rate of glove perforation and reported replacement of the perforated gloves during surgery with a similar glove [19, 22, 28]. But only one of the three studies reported that they only used the original gloves as data regardless of any further perforations in the replaced glove [22]. Only one study reported that the individual testing of the gloves was blinded to the allocation of the glove wearer [25]. 

One study included blinding of participants to condition [25], eight studies did not include blinding participants [5, 8, 9, 18, 19, 21, 23, 28], and five studies did not report blinding of participants to condition [20, 22, 24, 26, 27]. None of the studies reported participant refusal rates and withdrawal rates.

Selection issues were a potential source of bias for a number of studies. Thirteen of the fourteen studies were conducted in departments of surgery or emergency medicine [5, 8, 9, 18–24, 26–28], and nine studies focused on different surgical procedures [5, 8, 18–23, 27]. Only four studies evaluated interventions being used in different hospital units across different types of operations [9, 24, 26, 28], and only one study was conducted in a department of nursing [25]. The use of only surgical procedures included in these studies, and the homogeneity of individual participants may be a potential bias. Only two studies recruited the whole operation team including the principal surgeon, the surgical assistants, and the scrub nurses [18, 26]; eleven studies included either surgeons alone or both surgeon and first assistants [5, 8, 9, 18–24, 26, 28]. Because most of the studies tested the intervention of double gloving and blunt needles, gloves were collected immediately after each surgery. Only one study that was conducted in a department of nursing reported the consent rate and the dropout rate (86%) [25]. 

The studies differed in the method of comparing intervention and control groups which may bias interpretation of the results. Of the thirteen studies which aimed to compare the perforation rates [5, 8, 9, 18–24, 26–28], seven compared the gloves in the single-gloving condition separately with both the outer and inner gloves used in double-gloving conditions [5, 8, 18, 19, 21, 26, 28], two compared the inner gloves in both conditions [23, 27], two compared the total perforation numbers in both conditions [9, 24], one compared the number of surgical procedures with perforations [20], and one study did not report the method used [22]. Also the studies differed in a variety of ways, including the location of the intervention (country, hospital units), the time period of the study, and the number of participants. Four studies did not report the number of surgeons in the study instead reported the number of patients [5, 19–21], so it is possible that only a few surgeons generated most of the data which could limit the generalisability of the results.

### 3.6. Outcomes

The effectiveness of interventions using new devices to reduce needle-stick injuries in healthcare occupations was mainly defined by comparing the numbers of glove perforations or numbers of needle-stick injuries from the study populations with those of control populations. The effectiveness of educational training to reduce needle-stick injuries in healthcare occupations focused on comparing the changes in needle-stick injuries knowledge between study and control populations.

### 3.7. Double Gloving

Within the ten studies which evaluated double gloving or combinations of gloving [8, 9, 18, 19, 22–24, 26–28], seven compared single and double gloving [8, 9, 18, 19, 22–24, 26–28], two compared single, double, and combination gloving [23, 24, 27], and one compared one double-gloving method [23]. Eight studies reported an overall reduction by 9 to 15% in glove perforations in inner gloves under double-gloving conditions compared to those under single gloving conditions [8, 9, 18, 19, 22–24, 26–28]. One study only used descriptive statistics rather than inferential analysis to measure the differences of glove perforation rate [22]. One study, comparing two double-gloving methods with one, used two sterile surgical gloves and one used a nonsterile disposable inner glove under a sterile surgical glove [23], but did not find any significant statistical differences between groups. 

Of the four studies who reported the detection rate of glove perforation [9, 22, 24, 28], two reported a low detection rate in the double-gloving method [9, 20] and two reported a high detection rate when double gloving was used [24, 26]. The two studies which demonstrated a high detection rate differed from the two studies that found a low detection rate, in the brand of gloves used, which may be a factor in interpreting the results [9, 22, 24]. In these gloves, glove perforation during surgery results in an inflow of fluid between the two pairs of gloves. The wet area of the inner glove then appears as a bright green spot under the perforation area of the outer glove, which can be easily noticed by the wearer [24].

### 3.8. Blunt Needles

Three of the fourteen studies reviewed in this paper evaluated the use of blunt needles in reducing needle-stick injuries [5, 20, 21]. All of these studies were randomised control trials. Two studies reported a significant reduction by 9% to 16% in glove perforations for outer gloves of double gloving [20, 21], whereas one study did not find a significant difference in glove perforation between using blunt and sharp needles [5]. That study compared the rate of glove perforation for blunt and sharp needles used during obstetrical laceration repair which are said to require less time to complete [5], which may explain the apparent anomaly. The remaining two studies found a significant reduction in glove perforation when blunt needles were used during laparotomy [20] and Caesarean delivery [21]. The three studies reported the detection rate of glove perforation [5, 20, 21]. All of the three studies reported by surgeons that blunt needles were less convenient to use and associated with less satisfaction.

### 3.9. Educational Training

This quasiexperimental study examined the impact of structured training on prevention of occupational exposure to bloodborne pathogens on knowledge, behaviour, and incidence of needle-stick injuries among student nurses [25]. It reported a significantly higher score on both knowledge (*P* < .001) and behaviour (*P* = .002) in the group who received the bloodborne pathogens training. The self-reported needle-stick injuries were significantly lower for the bloodborne pathogens training group though they were not observed to practice universal precautions significantly more frequently than those in the control group [25].

## 4. Discussion

### 4.1. Main Findings

The reviewed interventions on needle-stick injuries includes components of double gloving, using blunt needles and educational training, and demonstrates there are significant reductions in needle-stick injuries following interventions as measured by glove perforations and changes in bloodborne pathogen knowledge. Results showed that interventions that use safeguard devices (double gloving and blunt needles) lead to a reduction in needle-stick injuries among healthcare workers. Knowledge regarding bloodborne pathogens was a major outcome measure in just one study [25] and results suggest that a structured bloodborne pathogen educational training program can lead to improvements in knowledge and a reduction in self-reported needle-stick injuries [25]. As such, the limited evidence regarding the effectiveness of educational training on bloodborne pathogen knowledge and behaviour was inconclusive. 

The results of this review are consistent with a much earlier review of interventions to prevent needle-stick injuries [6]. In this study, a reduction in the number of glove perforations was found in eight out of eleven studies. The current review included more recent studies in this field with more flexible study design (any case control studies) and types of interventions (double gloving, blunt needles and educational training). The current research findings are useful not only for encouraging healthcare workers to use double gloves and blunt needles during operations, but also for attracting policy makers to promote the universality of safeguards.

### 4.2. Limitation of the Reviewed Studies

The fourteen studies differed in a variety of ways, including the surgical procedures, the type of intervention, the location of the intervention (country, hospital units), the time period of the study, and the number of participants. Most studies reported relatively serious methodological flaws such as study procedure (13 studies), randomisation methods (5 studies), and statistical tests (10 studies). In addition, thirteen studies failed to report drop-out rate, blinding procedures, consent rate, and exact number of participants which may bias results [8, 9, 18, 19, 22–24, 26–28]. In the study conducted in a department of nursing, the possibility of communication between the study group and the control group may have influenced the effect of the educational training [25]. The lack of blinding may have introduced bias into the study as the participants in both groups knew the aim of the study and as a result may have paid more attention or been more careful during operations, which may decrease the function of the control group. Thus, caution about the differences within the fourteen studies is needed when drawing robust conclusions based on the results. 

Of the fourteen studies, only one examined the impact of a structured educational training program on prevention of occupational exposure to bloodborne pathogens on knowledge and behaviour among student nurses [25]. Although the other studies which investigated the effectiveness of safeguard devices have obtained significant results, a number of studies on the prediction of behaviours in health fields using social cognitive models suggest that intentions are the immediate antecedent to performing a specific behaviour [29]. In general, the stronger the intention to engage in behaviour is, the more likely it will be performed [29]. It is thought that if certain behaviours are planned to be performed in specified conditions (e.g., “I will wear double gloves and perform very carefully during the surgical procure”) and are consciously prepared (e.g., “set alarm”), when conditions are encountered the cues stimulate automatic activation of the behaviour [30]. In line with this evidence, it may be that a more effective intervention would contain structured educational training aimed at changing healthcare workers' attitudes and intentions to prevent needle-stick injuries, or combine educational training with a safeguards intervention, using implementation intentions. 

All the reviewed studies in this paper contained an experimental group and a control group, and most had proper randomised controlled procedures. However, it may raise ethical issues if interventions are designed in a randomised controlled manner which may put the healthcare workers at a high risk of incurring needle-stick injuries in the control group. The healthcare workers in the control group may have increased risk of experiencing needle-stick injuries compared to the healthcare workers randomised to the experimental group. Thus, such possible ethical issues need to be considered in order to minimise the risk for healthcare workers.

## 5. Conclusions

More studies are needed to evaluate interventions in nonsurgical settings, such as departments of nursing and other hospital units and among other healthcare personnel such as nurses. The current evidence suggests that both safeguard interventions and educational training programs are effective in reducing the risk of having needle-stick injuries. However, there are insufficient studies using a combination of both safeguards and educational interventions in surgical and nonsurgical settings. In future research, evaluations of these two types of interventions in both randomised controlled trials and in studies utilising other designs are needed.

## Figures and Tables

**Table 1:** Design of included studies.

Author	Design	Sample criteria	Sample size	Blinding
Punyatanasakchai et al. [18]	Randomized controlled trail	Patients and surgeons from Ramathibodi Hospital, Faculty of Medicine Mahidol University, Bangkok, Thailand	*N* = 300 (C: *n* = 150, I: *n* = 150)	No blinding

Kovavisarach and Seedadee [19]	Randomized controlled trail	Gynaecologic patients who underwent total abdominal hysterectomy (TAH) with or without bilateral salpingo-oophorectomy (BSO) at Rajavithi Hospital between September 1 1999 to August 31 2000. Primary surgeons and specialist assistants from the same hospital.	*N* = 170 (C: *n* = 82, I: *n* = 88)	No blinding

Nordkam et al. [20]	Randomized clinical trail	Patients who underwent laparotomy. Surgeons from the Department of Surgery	*N* = 200 (C: *n* = 100, I: *n* = 100)	Not reported

Wilson et al. [5]	Randomized prospective trial	Patients underwent an obstetric laceration repair in the labor and delivery suite from January 2005 through September 2006. Surgeons from Medical University of South Carolina	*N* = 438 (C: *n* = 221, I: *n* = 217)	No blinding

Sullivan et al. [21]	Randomized control trial	All patients requiring nonemergent cesarean deliveries from January to September 2006. Surgeons from Medical University of South Carolina	*N* = 194 (C: *n* = 97, I: *n* = 97)	No blinding

Caillot et al. [22]	Randomized control trial	Visceral surgical procedures performed in the surgical emergency department and 5 surgeons in the department.	*N* = 100 (C: *n* = 50, I: *n* = 50)	Not reported

Gaujac et al. [23]	Comparative randomized trial	Consecutive patients with maxillomandibular fractures. Two emergency room surgeons.	*N* = 42 (C: *n* = 47, I: *n* = 56)	No blinding

Laine and Aarnio [24]	Randomized prospective trial	All gloves used by the surgeons in 885 surgical operations at Satakunta Central Hospital.	*N* = 2462 (C: *n* = 1020, I: *n* = 1148, combination: *n* = 294)	Not reported

Wang et al. [25]	Quasiexperimental study	Students enrolled at Xiang Ya school of Medicine, Department of Nursing, in the 4-year nursing program	*N* = 106 (C: *n* = 50, I: *n* = 56)	Blinding

Naver and Gottrup [26]	Randomized control study	Gloves tested on surgeons, assistants and scrub nurses in university hospital, Denmark	*N* = 566 (C: *n* = 306, I: *n* = 260)	Not reported

Thomas et al. [9]	Randomized control study	Surgical procedures lasting more than one hour performed in department of general surgery, lady hardinge medical college and associated srimati sucheta kriplani hospital, New Delhi.	*N* = 396 (C: *n* = 198, I: *n* = 198)	No blinding

Laine and Aarnio [27]	Randomized control study	All gloves used inconsecutive orthopaedic and trauma operations, conventional and arthroscopic in Satakunta central hospital, Pori, Finland	*N* = 972	Not reported

Lancaster and Duff [8]	Cohort study	Gloves from obstetric and gynaecologic surgical procedures at University of Florida College of Medicine	*N* = 100 (C: *n* = 325, I: *n* = 675)	No blinding

Na'aya et al. [28]	Cohort study	Gloves used in general surgical procedures in department of surgery, University of Maiduguri teaching hospital, Nigeria.	*N* = 1120 (C: *n* = 240, I: *n* = 880)	No blinding

**Table 2:** Intervention design and findings.

Author	Consent rate (CR)/ Response rate (RR)	Intervention type	Surgical procedure	Randomization procedure and design	Study timeline	Outcome measure	Results
Punyatanasakchai et al. [18]	Not reported	Double gloving	Episiotomy repair after vaginal delivery	The surgeons were randomly selected one of two envelopes, number 1 representing the single-gloving method and number 2 representing the double-gloving method.	7 months	Glove perforation rate, duration of operation, position level of surgeons	No significant difference in the frequency of perforations between the double-outer gloves (22.6%) and single-gloves (18%). A significant reduction in glove perforations between the double-inner gloves (4.6%) compared with the single-gloves (18%) (*P* < .05).

Kovavisarach and Seedadee [19]	Not reported	Double gloving	Gynaecological surgery	Primary surgeons were randomly allocated to use either the single-gloving or the double-gloving method.	12 months	Glove perforation rate, duration of operation	A significant difference was found in the glove perforation rate between double-inner glove (6.09%) and single gloving group (22.73%). No significant difference between the glove perforation rates in single gloves (22.73%) and in double-outer gloves (19.5%).

Nordkam et al. [20]	Not reported	Blunt needle	Abdominal wall closure	Surgeons were randomised by envelop to use either blunt needle or sharp needle	6 months	Glove perforation rate, evaluation of the blunt needle	A significantly higher number of surgical procedures with perforations using the sharp needle (*P* = .003) than with the blunt tapered needle. Detection rate was low (21%). Blunt tapered needles are less convenient

Wilson et al. [5]	Not reported	Blunt needle	Obstetrical laceration repair	Patients with obstetric lacerations were randomized to repair with either blunt or sharp needles.	21 months period	Glove perforation rate, evaluation of the blunt needle, and position level of surgeons	No significant difference in the glove perforation rate between blunt and sharp needles. There was poor correlation between reported perforations and those detected by water test. Blunt needles were reported more difficult to use (*P* = .0001)

Sullivan et al. [21]	Not reported	Blunt needle	Cesarean-delivery closure	Patients requiring cesarean delivery were assigned randomly to receive closure with either blunt or sharp needles	21 months	Glove perforation rate, evaluation of the blunt needle, and duration of operation	A significant reduction in total glove perforation rate for the primary surgeon with blunt needles (7.2%) compared with sharp needles (17.5%) as well as for the assistant surgeons. Poor correlation between reported perforations and those detected by water test. Physicians reported low satisfaction with blunt needles compared with sharp needles (*P* < .001)

Caillot et al. [22]	Not reported	Double gloving	Visceral surgical procedures	Visceral surgical proceduresperformed in the Surgical Emergency Departmentwere randomly assigned todouble gloving or single gloving	3 months	Glove perforation rate, detection of the perforation, duration of operation	Did not adequately compare the rate of glove perforation. Double gloving allowed significantly higher detection rates of glove perforation (*P* < .001)

Gaujac et al. [23]	Not reported	2 types of double gloving	Arch bar placement	Patients were equally divided into 2 groups. In group 1, 2, sterile surgical gloves were used; in group 2, a nonsterile disposable inner glove was used under a sterile surgical glove.	Not reported	Glove perforation rate, duration of operation	No significant statistical difference was found between 2 double gloving methods in terms of inner glove perforations

Laine and Aarnio [24]	Not reported	Double gloving	General surgical operations	Patients born in even years were operated on with double gloving and those born in uneven years were operated on with single gloving	2 months	The glove type, the operating time, the type of surgery, the detection rate and location of perforation	A low number of perforations of the inner glove of the double-gloving system were detected. Higher detection of perforation in double-glove system (*P* < .001). The longer of the operating duration, the higher rate of perforation

Wang et al. [25]	RR: 86%	Educational training	NA	One class was randomly assigned to receive the educational intervention, and the other served as a comparison group, receiving standard education.	4 months	Changes in knowledge and self-reported universal precautions behaviour, observed adherence to universal precautions, and self-reported needlestick injuries	The group that received the intervention scored significantly higher than the standard education group on both knowledge (*P* < .001) and behaviour (*P* = .002), and were less likely to experience needlestick injuries (*P* = .004)

Naver and Gottrup [26]	Not reported	Double gloving	Various types of gastrointestinal surgery	The surgeons, assistants and scrub nurses were randomized into one of two groups. In group one the operating team was using powder-free single gloves and group two used a powder-free double-gloving system.	Not reported	Glove perforation rate, detection of the perforation, and the position of the participants	A significant difference between single gloves and inner indicator gloves (*P* < .005). The surgeon in indicated gloving group obtained high detection rate of glove perforation (*P* < .0001)

Thomas et al. [9]	Not reported	Double gloving	General surgical operations	The gloving pattern was randomized into two groups of the equal number by sealed envelopes	Not reported	Glove perforation rate, detection of the perforation, evaluation of double gloving	In double-gloving pattern, 32 glove perforations were observed, of which 22 were in the outer glove and 10 in the inner glove. Majority of glove perforations (83.3%) went unnoticed. Double gloving was accepted by majority of surgeons.

Laine and Aarnio [27]	Not reported	Double gloving	Orthopaedic and trauma surgery	Before the operations, the surgeons were randomised to use either single gloves, double indicator gloves or a combination of two regular surgical gloves on top of each other	2 months	Glove perforation rates, detection of perforations, operation types, and duration of operation	Significant difference in perforations of the inner glove in two of indicator gloves and in the regular combination gloves when the outer glove was perforated (*P* = .02)

Lancaster and Duff [8]	Not reported	Double gloving	Obstetric and gynecologic surgical procedures	The choice to single versus double glove was left to the discretion of the individual surgeon.	7 months	Glove perforation rate, the association between position of the surgeon and perforation rate	11% of single glove sets contained a perforation whereas only 2% of double glove sets contained a corresponding defect in the inner and outer gloves (*P* < .01)

Na'aya et al. [28]	Not reported	Double gloving	General surgical procedure.	The surgeons wore single or double gloves at their own discretion.	Not reported	Glove perforation rate, detection rate of the perforation, and duration of operation	A significant greater risk for blood-skin exposure in the single glove sets (*P* < .01) Most perforations were not noticed during the surgery.
